# Ultra-Fast Warming Procedure of Vitrified Blastocysts Results in Maintained Embryology and Clinical Outcomes

**DOI:** 10.1007/s43032-024-01762-x

**Published:** 2025-01-09

**Authors:** Jenna Lammers, Arnaud Reignier, Sophie Loubersac, Maxime Chaillot, Thomas Freour

**Affiliations:** 1 Service de Médecine Et Biologie de La Reproduction, Hôpital Mère Et Enfant, CHU de Nantes, 38 Boulevard Jean Monnet, Nantes, France; 2 Nantes Université, CHU Nantes, INSERM, Center for Research in Transplantation and Translational Immunology, UMR 1064, 44000 Nantes, France

**Keywords:** Blastocyst, Vitrification, Warming, IVF, Embryology, Clinical outcome

## Abstract

Vitrification has revolutionized embryo cryopreservation, but represents a significant workload in the IVF lab. We evaluated here an ultrafast blastocyst warming procedure in order to improve workflow while maintaining clinical outcome. We first evaluated the expression of main markers of lineage specification in a subset of blastocysts donated to research warmed with ultrafast protocol. We then performed a prospective pseudo-randomized pilot study comparing blastocyst survival, reexpansion and live birth rates between standard (3 steps, 15 min), and ultrafast warming protocol (1 step, 2 min). Finally, survival, reexpansion and live birth rates (LBR) obtained with ultrafast warming protocol were prospectively collected during 3 months and compared with previous indicators. Immunofluorescence experiments showed that staining and spatial organization of cell fate markers were conserved with ultrafast protocol. Survival, reexpansion and LBR were strictly comparable between standard (n = 47 cycles) and ultrafast (n = 39 cycles) groups in the pilot study (100 vs 100%, 80 vs 76% and 29.8 vs 30.7% in standard and simplified groups respectively). Survival, expansion and LBR obtained with the ultrafast warming protocol over the next 3-month period (321 cycles, 336 embryos) were comparable with those obtained with the standard protocol throughout the 6 months (547 FBT cycles, 578 embryos) preceding shifting protocol (97.6 and 29.6% vs 97.8 and 28.3% respectively, p > 0.05 for both). In conclusion, using an ultrafast blastocyst warming procedure results in similar embryology and clinical outcomes compared with standard protocol, but significantly shortens the technical procedure, ultimately improving the overall lab’s workflow.

## Introduction

Cryopreservation has progressively become central in ART over the last decades, both for oocytes and embryos. Elective embryo cryopreservation has extended from supernumerary ones to freeze-all strategies, for instance in case of risk of ovarian hyperstimulation syndrom (OHSS) or after trophectoderm biopsy at the blastocyst stage, reaching 35.5% of all ART cycles in 2018 in Europe, as compared to 15% 10 years before in 2008 [1]. Since the first pregnancy after frozen-thawed embryo transfer in 1983, freezing protocols have largely evoluted, shifting from in-house media and protocols for slow freezing to commercial media with precise cryoprotectant composition and standardized protocol for vitrification. The use of vitrification for embryo cryopreservation rapidly replaced slow-freezing over the last 2 decades. In brief, vitrification is based on the use of high concentrations of cryoprotectants and ultra-rapid cooling or warming rates leading to the solidification of cells into a glass-like state without ice formation. This technique revolutionized cryopreservation in ART cycles, with very significantly higher oocyte and embryo survival rates, and ultimately higher success rates than with slow-cooling [2]. However, the whole vitrification and warming procedures take time, with successive steps of well-defined duration which must be respected by the operator.

In this context of rapidly increasing use of vitrification in IVF labs with heavy workload, the issue of time spent for technic and time-effectiveness of vitrification and thawing protocols has logically been raised. Most vitrification or thawing protocols last 15–20 min. Although this can be slightly optimized by performing several cycles simultaneously, this remain somewhat technically risky and should probably not be recommended. Concerning vitrification step, some semi-automated systems were recently launched, aiming at standardizing the process and improving time-effectiveness. However, the currently available evidence questions not only their superiority over manual method in terms of clinical outcome, but also their time-effectiveness, as the overall duration of the procedure does not appear to be significantly reduced [3]. Moreover, these systems are not designed to handle warming process. The manufacturers’ recommendation for blastocyst warming after vitrification consist in successive exposition to decreasing concentrations of cryoprotectants. However, it can be speculated that the majority of cryoprotectants is removed during first step of warming, questioning the usefulness of using the following successive steps. This, if validated, could ultimately result in significantly shorter warming procedures. Some teams very recently reported such pilot studies on shortened warming protocol with encouraging embryology outcomes in terms of embryo survival and re-expansion rates [4–7], but the clinical outcomes were not evaluated. Moreover, the implementation of a new protocol of embryo warming should not only focus on embryo morphology and ability to implant, but also on molecular aspects of embryo development and cell specification. In this context, it might be relevant to also evaluate the expression of cell type and fate markers at the blastocyst stage, in order to confirm that the overall architecture and cell lineage are conserved when changing the warming procedure.

In this study, we first evaluated the expression of cell lineage markers in blastocysts donated for research and warmed with ultrafast protocol. We then conducted a pseudo-randomized prospective pilot study followed by a cohort study comparing embryology and pregnancy outcomes after frozen-thawed blastocyst transfer according to the warming method used, i.e. standard or ultrafast simplified procedure.

## Materials and Methods

This study combined basic research and a clinical study.

### Basic Research Experiments

First, the basic research experiments were conducted in May 2022. Survival and expansion of blastocysts donated for research and warmed with ultrafast protocol (see below) were recorded. Embryos were then stained for cell specification markers. The use of human embryo donated to research as surplus of IVF treatment was allowed by the French embryo research oversight committee: Agence de la Biomédecine, under approval number RE13-010 and RE18-010. Immunofluorescence experiments were then performed for GATA4 (marker of primitive endoderm), NR2F2 (marker of trophectoderm) and NANOG (marker of epiblast). The number and spatial repartition of the positive cells were recorded for each marker in order to evaluate lineage specification, as reported before by our team [8].

### Clinical Study and Ethical Approval

#### Study Design

Second, the prospective monocentric clinical pilot study was conducted in a University-based IVF centre in July 2022, and aimed at comparing embryology and clinical outcome after frozen-thawed blastocyst transfer according to warming protocol, i.e. standard (group 1) or ultrafast (group 2). The design was pseudo-randomized, as patients were alternately allocated to one group or another on a weekly basis. Data obtained after implementation of the simplified protocol were then prospectively collected in an observational study during the following 3 months (August-October 2022) and compared with those obtained throughout the 6-month period (January-June 2022) before shifting procedure.

#### Ethics

Data were anonymously collected from the local database, in accordance with the French National Commission for Information and Liberties (CNIL). All patients gave their consent for the anonymous use of their clinical data. The protocol was approved by local ethics committee (GNEDS) (decision 23–117-09–250).

#### Embryo Culture and Freezing Strategy

ICSI or IVF was chosen according to sperm quality and couple’s history. All embryos were systematically cultured in a time-lapse device (Embryoscope® + , Vitrolife, Sweden) up to the blastocyst stage in a single step culture media (G-TL®, Vitrolife, Sweden) under low oxygen atmosphere (6% CO2, 5% O2) from insemination/injection onwards. Each blastocyst was evaluated according to Gardner and Schoolcraft’s classification [9] on day 5 (115 ± 1 h), and if applicable on day 6 (139 ± 1 h) (Gardner et al., 2000). Supernumerary day 5 blastocysts could be frozen from B3 to B6 except those with grade C trophectoderm. When blastocyst quality did not reach these criteria, culture was prolonged up to day 6, where only ≥ B4BB could be vitrified. Blastocysts were vitrified with Rapid Vit Blast® kit (Vitrolife, Sweden) according to manufacturer’s instructions, and stored in liquid nitrogen.

#### Embryo Thawing

Embryo warming was performed at least 4 h before transfer and blastocyst re-expansion and viability were systematically evaluated approximately 2 h after thawing. In the standard protocol group (group 1), blastocysts were thawed with Rapid Warm Blast® kit (Vitrolife, Sweden) according to manufacturer’s instructions. In brief, 3 successive media are used at 37 °C, with Warm 1 Blast (2 min) and Warm 2 Blast (3 min) media containing medium and low sucrose levels respectively. Embryos were then washed in Warm 3 blast (not containing any cryoprotectant) during 10 min, before being moved to the conventional culture medium (G-TL, Vitrolife, Sweden). The overall procedure took 15 min. Embryos were then cultured during 4–5 h (at 37 °C, 6%CO2, 5%O2) before transfer. Re-expansion was evaluated before transfer. In the ultrafast protocol group (group 2), blastocysts were thawed with the same kit, but only step 1 was used, with embryos cultured 2 min in Warm 1 Blast medium and then directly moved to the conventional culture medium. In that case, the duration of the overall procedure was 2 min. Further culture and transfer procedure was similar.

#### Frozen-Thawed Blastocyst Transfer Cycles

All patients underwent an artificial cycle with hormonal replacement therapy. Endometrial thickness ≥ 7 mm with trilaminar aspect was mandatory before transfer. Estrogen therapy was continued and combined with vaginal micronized progesterone (400 mg daily) for 6 days before embryo transfer. Doses were increased after transfer (estrogen 8 mg per day and micronized progesterone 800 mg per day).

#### Cycle Outcome

Embryology outcome were embryo survival rate and embryo expansion rate, defined as the proportion of embryos fully recovering their pre-vitrification expansion within 2–3 h after warming. Main clinical outcome was live birth rate, as defined by delivery of a live newborn after 22 weeks of pregnancy. Clinical pregnancy rate was also recorded and defined as the proportion of cycles with fetal heart beat at early ultrasound (6–7 weeks of pregnancy). Embryology and clinical outcomes were compared between both groups according to thawing procedure.

## Results

### Basic Research Experiments

**Fig. 1:**
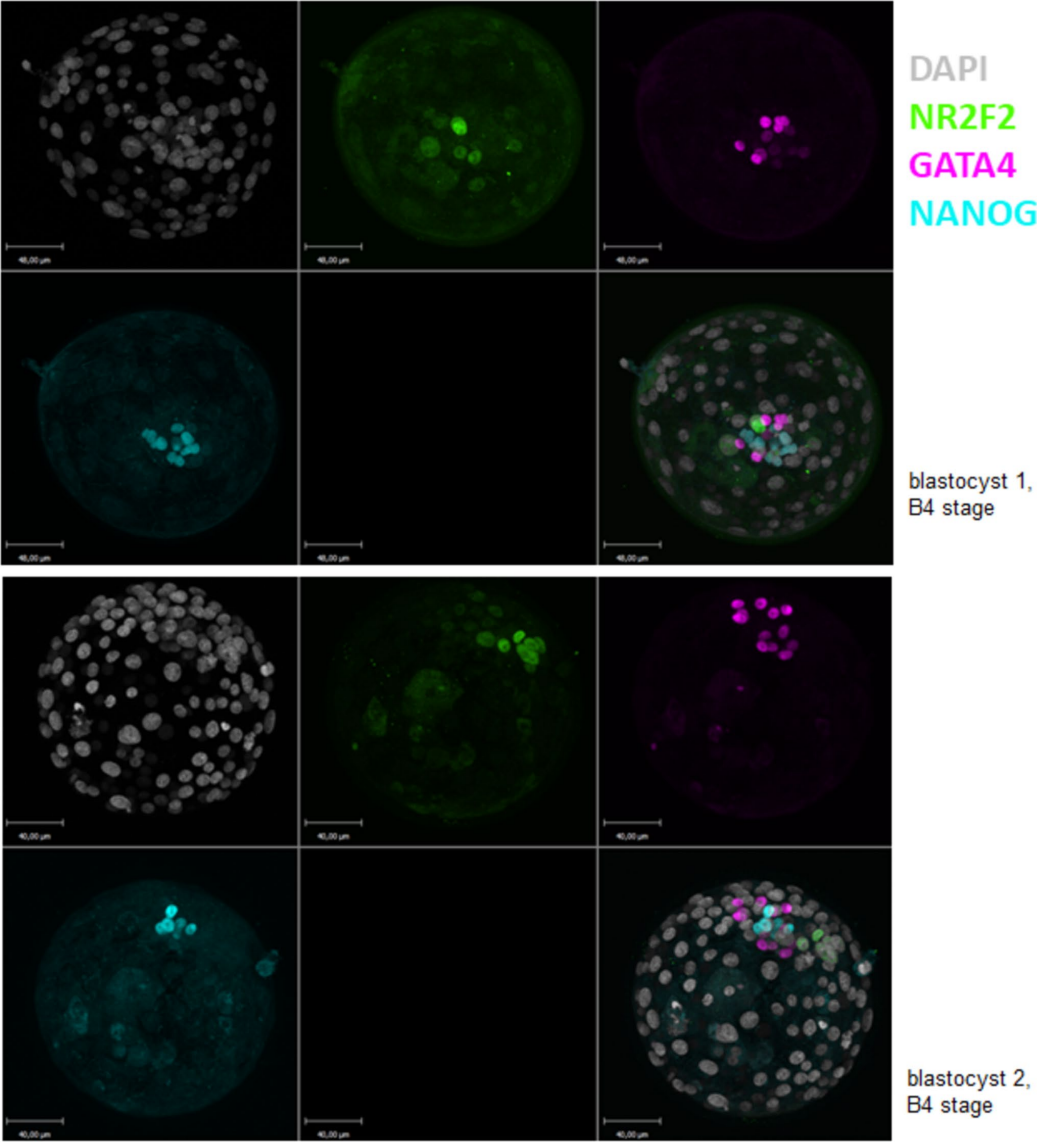
Immunofluorescence of NR2F2 (green), GATA4 (purple) and NANOG (cyan) at the B4 blastocyst stage (2 embryos). Nuclear counterstaining is white

A total of 27 blastocysts (24 D5 and 3 D6) donated for research were warmed with the ultrafast protocol, leading to 100% survival and 82% reexpansion rate. Among them, 21 were fixed for Immunofluorescence and imaging. The expression of GATA4, NR2F2 and NANOG is presented in Fig. 1. Distinct GATA4 and NANOG positive cells can be observed in the inner cell mass (ICM). NR2F2 positive trophectoderm cells can be seen in polar trophoblast, just in contact with ICM. Overall, the proportion and localisation of positive cells for each markerweare consistent with what was reported before by our team in blastcysts warmed with standard protocol [9].

### Pseudo-Randomized Pilot Study

A total of 92 blastocysts were included in the study. In group 1 (standard protocol), 52 blastocysts (45 D5 and 7 D6) were warmed in 47 FBT cycles. Survival and re-expansion rates were 100% and 80% respectively. No transfer was cancelled. Clinical pregnancy rate was 36.2% and LBR was 29.8%. In group 2 (ultrafast protocol), 42 blastocysts (36 D5 and 6 D6) were warmed in 39 FBT cycles. Survival and re-expansion rates were 100% and 76% respectively. No transfer was cancelled. CPR was 38.4% and LBR was 30.7% (Table 1).

**Table 1:** Outcomes of the pseudo-randomised pilot study

	Group 1 Conventional warming protocol (n=47 FBT cycles, 52 embryos)	Group 2 Ultrafast warming protocol (n=39 FBT cycles, 42 embryos)	*p*
Survival rate (%)	100	100	>.05
Expansion rate (%)	80	76	>.05
Clinical pregnancy rate (%)	36.2	38.4	>.05
Live birth rate (%)	29.8	30.7	>.05

Results are presented as mean±standard deviation or proportion where appropriate

### Prospective Cohort Study

The ultrafast warming protocol was implemented in the lab in August 2022. Survival and LBR during the following 3-month period (Aug-Oct 2022, n = 336 embryos, 321 FBT cycles) were comparable to those observed during the 6-month period of time preceding the shift in warming protocol (January-June 2022, n = 578 embryos, 547 FBT cycles) (Table 2).

**Table 2:** Outcomes of the prospective cohort study

	Group 1 Conventional warming protocol (Jan-Jun 2022, n=547 FBT cycles, 578 embryos)	Group 2 Ultrafast warming protocol (Aug-Oct 2022, n=321 FBT cycles, 336 embryos)	*p*
Female age (years)	34.2±4.9	34.3±4.8	>.05
BMI (kg/m²)	24.9±5.4	25.0±5.5	>.05
Active smoker (%)	16.8	15.6	>.05
Primary infertility (%)	59	59.6	>.05
ICSI (%)	61.5	58.9	>.05
Day 5 blastocysts (n, %)	90.1	87.8	>.05
Single embryo transfer (%)	95.2	95	>.05
Survival rate (%)	97.6	97.8	>.05
Expansion rate (%)	80.5	80.3	>.05
Biochemical pregnancy rate (%)	41	42	>.05
Pregnancy loss rate (%)	29.9	29.6	>.05
Clinical pregnancy rate (%)	38.2	39.2	>.05
Live birth rate (%)	28.7	29.6	>.05

Results are presented as mean±standard deviation or proportion where appropriate. Biochemical pregnancy was defined by beta hCG serum level >100 IU/L. Pregnancy loss rate was defined by the proportion of biochemical pregnancies not leading to live birth

## Discussion

In this pilot study, we demonstrated that warming blastocysts with an ultrafast 1-step warming protocol results in comparable embryology and clinical outcomes compared with conventional 3-step warming protocol. These encouraging indicators were maintained throughout the 3-month follow-up period at the same level than usually observed during previous period with conventional protocol. We also showed that the expression of main cell fate markers at the blastocyst stage remain unchanged with this new simplified warming protocol.

Vitrification was initially developed in the 90’s in order to overcome intracellular and extracellular ice crystal formation and subsequent poor survival rates in oocytes and embryos due to incomplete dehydration observed with slow freezing. Since then, vitrification demonstrated its superiority over slow-freezing in terms of clinical outcomes and cryosurvival rates for oocytes, cleavage-stage embryos and blastocysts, as elegantly reviewed in [2]. Biologically, the success of vitrification relies on ultrafast colling temperature and high concentration of cryoprotectant agent in order to skip ice formation and reach a vitreous, glass-like cellular state [10, 11]. Whatsoever the brand and the cryoprotectant molecule considered (DMSO, ethyleneglycol), the high concentration of cryoprotectant agent is reached progressively to allow cells achieve deep dehydration while maintaing structural integrity. Interestingly, it has been demonstrated that the intracellular concentration of cryoprotectants after vitrification was lower than after slow freezing despite exposure to higher concentration of cryoprotectant solutions [12]. Thawing and warming of cells has often been considered as less critical than cooling, and has therefore been less investigated. Overall, the same (but inverse) approach than for vitrification was used during warming to progressively remove cryoprotectant agent and rehydrate embryonic cells or oocyte, exposing them to successive media with decreasing sucrose concentration. This historical belief that multi-step cell recovery is preferable might originate from very early work on freezing/thawing which did not use sucrose but only decreasing concentrations of cryoprotectant, hence making stepwise thawing mandatory [13].

Actually, if not closely controlled, the exposure of cells to osmotic stress during the thawing process can result in the alteration of microtubules, organites, membrane and and ultimately cell lysis [10]. Warming rate has been explored in several studies, pointing at the importance of reaching the highest warming rate possible. Some authors even suggested that warming rate is significantly more critical for cell survival than cooling rate [14, 15]. In this context and quite surprisingly, the duration of exposure to warming solution(s) with decreasing CPA concentrations has hardly ever been reported in the literature. Actually, various CPA concentration and exposure time have been proposed, but very little data are available and evidence seem to be still missing. However, it can be speculated that the vast majority of cryoprotectant removal occurs rapidly, potentially questionning the relevance of culturing the embryo in successive baths with decreasing CPA concentration during warming procedure. The lower intracellular concentration of cryoprotectants found after vitrification than after slow freezing already cited above supports this reflexion [12]. Furthermore, high sucrose level, and subsequent concern on osmotic stress, might not be mandatory for blastocyst warming, as reported in early work by Lane and Gardner [16], where they demonstrated that warming could be performed efficiently with low sucrose concentration (0.25 M), instead of 1 M concentration as reported in various studies [17]. Therefore, the combination of of less intracellular CPA for vitrification, together with the experience of warming with low sucrose level might support the use of single step warming protocols for blastocysts.

In this context, a few authors recently proposed the use of a simplified warming protocol and reported preliminary data in various congresses during the last months. Manns et al. [6] reported a prospective randomized study conducted in 71 donated blastocysts, warmed with either ultra-fast (single-step) or standard protocol. No significant difference in terms of survival and re-expansion was found between the 2 protocols. Naert and al [4] reached the same conclusion when comparing survival and re-expansion rates of biopsied and non biopsied blastocyst with either ultra-fast or standard warming protocol, highlighting that neither the blastocyst stage at vitrification nor the biospy status affected these results. Interestingly, Guns & Ahlstrom [7] compared 2 different sucrose concentrations for ultrafast warming protocol. Both low and high sucrose concentrations yielded high survival and expansion rates, comparable to the standard procedure. Altogether, these 3 reports are in agreement with our results and confirm that ultrafast warming protocol does not affect blastocyst survival and re-expansion rates, and suggest that ultrafast warming protocol can be used with various sucrose concentrations at various temperature (i.e. 37 °C or room temperature).

We believe that this pilot study has some strengths. First, and as far as we know, it is the first of its kind with a pseudo-randomised design. Second, the prospective follow-up on a large sample of cycles brought reassuring clinical information on the clinical relevance and safety of this new simplified warming protocol. Third, the combination of a clinical trial with some more fundamental experiments brings more insight into the expected safety of the procedure. We however acknowledge that this pilot study also has some limitations. The limited number of cycles included the pseudo-randomized study advocates for further confirmation in larger well-designed RCTs, with pregnancy and delivery follow-up, including neonatal aspects. The monocentric design and the use of one brand of vitrification / warming media also prevent from drawing general conclusion in other settings and/or with other vitrification / warming media.

In conclusion, we report here some encouraging preliminary data on a simplified warming protocol for vitrified blastocysts. Our results illustrate that this protocol appear to perform equally well as conventional warming procedure, but with much shorter technical time, hence being a great opportunity for increasing flexibility and time-efficiency in busy IVF labs with heavy workload.

## Data Availability

Data not publicly available but reasonable requests can be sent to Correspinding author.
